# Circulating cell-free nucleic acids of plasma in human aging, healthy aging and longevity: current state of knowledge

**DOI:** 10.3389/fgene.2023.1321280

**Published:** 2023-11-28

**Authors:** Nicolas P. Tessier, Lise M. Hardy, Jean-François Deleuze, Alexandre How-Kit

**Affiliations:** ^1^ Laboratory for Genomics, Foundation Jean Dausset—CEPH, Paris, France; ^2^ Centre National de Recherche en Génomique Humaine, CEA, Institut François Jacob, Evry, France

**Keywords:** plasma, circulating cell-free nucleic acids, circulating cell-free DNA, circulating cell-free miRNA, aging, longevity, healthy aging, centenarian

## Abstract

Circulating cell-free nucleic acids (ccfNAs) of plasma are a remarkable source of genetic, epigenetic and transcriptomic materials originating from different cells, tissues and organs of an individual. They have been increasingly studied over the past decade as they can carry several important pieces of information about the health status of an individual, which makes them biomarkers of choice for non-invasive diagnosis of numerous diseases and health conditions. However, few studies have investigated variations of plasma ccfNAs in healthy subjects, particularly in relation to aging, healthy aging and longevity, despite the great variability of these biological processes among individuals. Here, we reviewed several studies that focused on the analysis of circulating cell-free DNA (ccfDNA) and microRNAs (ccfmiRNAs) during aging and in the elderly, including some on exceptionally long-lived individuals, i.e., centenarians. After a brief overview of the types, origins and functions of plasma ccfNAs, we described the variations of both ccfDNA and ccfmiRNAs during aging as well as the identification of several potential ccfDNA-based and ccfmiRNA-based biomarkers of aging, healthy aging and/or longevity. We finally highlighted some prospects offered by ccfNAs for the understanding and improvement of healthy aging and longevity.

## 1 Introduction

Human aging is a time-dependent biological process that leads to an increased susceptibility toward frailty and death through progressive decline of physical and cognitive capabilities associated to the alteration of several molecular, cellular and physiological functions (Lopez-Otin et al., 2013; 2023). Aging also differentially affects individuals, who can present considerable differences in both their lifespan and healthspan. Among them, centenarians and supercentenarians represent the upper limit of these phenotypes (Pignolo, 2019). To date, despite their great potential in this field, few studies have specifically focused on the analysis of blood plasma ccfNAs and their inter-individual variability in healthy subjects, notably in the context of aging, healthy aging and longevity.

Noteworthy, ccfNAs have been primarily investigated as biomarkers for non-invasive diagnosis in oncology and prenatal screening from liquid biopsies, following the discovery of circulating cell-free tumoral and fetal DNA in the bloodstream (Tong and Lo, 2006). Their study has been greatly facilitated through the development and improvement of highly sensitive and/or genome-wide analytical technologies, including real-time and digital PCR as well as next-generation sequencing (NGS) (Lewis et al., 2015). As a consequence, their translational and clinical potentials have been increasingly assessed in many human diseases since more than a decade (Bottani et al., 2020; Medina et al., 2023).

In this mini-review we have summarized the state of knowledge of plasma ccfNAs in human aging and longevity, including a first part on the description and origin of ccfNAs in healthy individuals, followed by the description of variations of circulating cell-free DNA (ccfDNA) and micro-RNA (ccfmiRNA) during aging and in relation to healthy aging and longevity. We finally concluded by mentioning some perspectives offered by ccfNAs for the understanding and improvement of healthy aging and longevity.

## 2 An overview of the biology and applications of plasma ccfNAs

**FIGURE 1:**
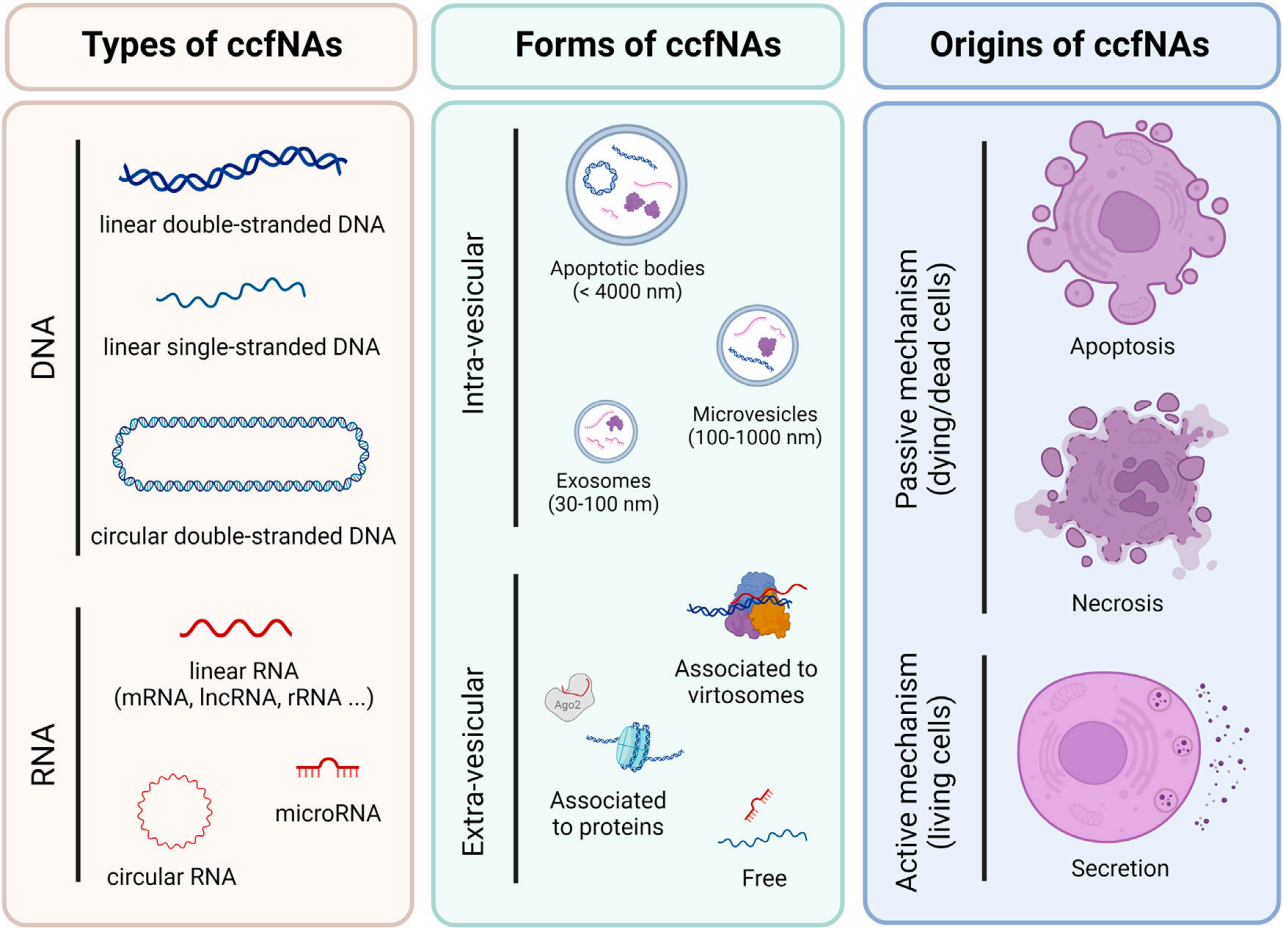
Overview of different types, forms and origins of circulating cell-free nucleic acids in plasma. The list is not exhaustive but corresponds to the most and best described.

Endogenous ccfNAs can be divided into circulating cell-free DNA (ccfDNA) and RNA (ccfRNA). To date, ccfDNA is the most studied type of ccfNAs. It can be of nuclear (ccfnDNA) or mitochondrial origin (ccfmtDNA), single or double-stranded, and in linear or circular form (Figure 1). ccfRNA encompasses coding (mRNA) and non-coding RNA (long non-coding RNA, circular RNA, and small RNA). Among these, miRNAs are currently the most studied type of ccfRNA (Suarez et al., 2022). Plasma ccfNAs can be contained within extra-cellular vesicles (EV), which are composed of apoptotic bodies (<4,000 nm), microvesicles (100–1,000 nm) and exosomes (30–100 nm) and can also be bound to proteins (histones, Argonaute proteins, etc.) and/or macromolecular structures, notably virtosomes (Figure 1) (Tzimagiorgis et al., 2011; Thierry et al., 2016; Aucamp et al., 2018).

The forms associated to ccfNAs are directly related to their origins and several active and passive mechanisms have been proposed for their release into the bloodstream, including apoptosis, necrosis and secretion, which also affect the size of ccfDNA. Thus, small fragments (≈150 nt) would be derived from apoptosis and large fragments (≥1,000 nt) from necrosis and secretion (Figure 1) (Tzimagiorgis et al., 2011; Aucamp et al., 2018; Grabuschnig et al., 2020). The main cellular contributors to plasma ccfNAs differ according to their types or sometimes to the method used for deconvolution. DNA methylation analyses showed that plasma ccfDNA mainly originated from leukocytes, erythrocyte progenitors and vascular endothelial cells (Moss et al., 2018), whereas nucleosome footprints identified lymphoid and myeloid cells as their main contributors (Snyder et al., 2016). More recently, monocytes, neutrophil and bone marrow megakaryocytes were also highlighted from ChIP-seq experiments as major sources of ccfDNA (Sadeh et al., 2021). Regarding ccfRNA, RNA-seq data identified platelets, erythrocyte progenitors and leukocytes as the main contributing cells (Vorperian et al., 2022). Additionally, RT-qPCR experiments highlighted that plasma ccfmiRNAs were mainly released by white and red blood cells (Pritchard et al., 2012).

Although still under debate and poorly documented, there are several suggested putative biological functions of ccfNAs, which vary according to their types and forms. ccfDNA has initially been thought of as cellular waste with little to no biological function, but are now increasingly seen as mediators of cell-to-cell communication. Thus, they have been proposed as putative pro-inflammatory molecules when recognized as DAMP (danger-associated molecular patterns) by immune cells, notably unmethylated ccfmtDNA fragments. They are also considered as mediators of (epi)genetic homeostasis, genometastasis, damages, apoptosis and/or antitumoral activities after penetration into a cell, when bound to proteins or associated to virtosomes (Thierry et al., 2016; Aucamp et al., 2018). In contrast, ccfRNA functions are thought to be mostly carried by ccfmiRNAs contained in exosomes and micro-vesicles, which could serve as long distance intercellular messengers after delivery to the targeted cells, acting on gene silencing through mRNA transcript degradation (Mathieu et al., 2019).

Applications of plasma ccfNAs mainly involve their use as non-invasive diagnostic biomarkers in diseases, in particular cancer. The concentration, sequence, copy number variations, integrity and methylation of ccfDNA as well as expression signature of ccfmRNAs and ccfmiRNAs have been proposed as diagnostic biomarkers in several diseases (Armand-Labit and Pradines, 2017; Bruno et al., 2020). Another application for ccfmiRNAs includes their use as therapeutic agents in diseases (Pozniak et al., 2022).

## 3 Variations of ccfDNA during aging and potential biomarkers of healthy aging

Data regarding quantitative and qualitative modifications in ccfDNA associated with aging, healthy aging and/or longevity are scarce. The following describes studies focusing on the analysis of plasma ccfDNA in aging and the elderly, sometimes considering its nuclear and mitochondrial origin (Table 1).

**TABLE 1:** Potential plasma ccfNAs-based biomarkers of aging, healthy aging and/or longevity.

Types of ccfNAs	Description	Methods used	Study design	Biomarker of^1^			Observations	References
				Aging	Healthy aging	Longevity		
ccfDNA	Total/nuclear ccfDNA concentration	qPCR	18–69 y.o. (n = 104)	++			↑ with age	Meddeb et al. (2019)
		qPCR	22–37 y.o. (n = 12) and ≥90 y.o. (n = 8)			+	↑ in nonagenarians	Jylhava et al. (2011)
		QuantiFluor^®^ dsDNA System (Promega)	65–98 y.o. (n = 101)		+	+	No difference with age but ↓ with exercise and nutritional supplementation	Tosevska et al. (2016)
	ccfmtDNA concentration	qPCR	20–69 y.o. (n = 78)	+			↑ with age^2^	Padilla-Sanchez et al. (2020)
			1–104 y.o. (n = 831)	+++			↑ with age in adults	Pinti et al. (2014)
			30–64 y.o. (n = 67)	++			↓ with age when enclosed by extra-cellular vesicles	Lazo et al. (2021)
	*LINE-1* methylation	MS-PCR	18–64 y.o. (n = 62)	+++			Hypomethylation in the older group (41–64 y.o.)	Erichsen et al. (2018)
	*Alu* methylation	MS-PCR	18–64 y.o. (n = 62)	+			Hypomethylation in the older group (41–64 y.o.)	
	Nucleosomal ccfDNA signal	Whole ccfDNA sequencing (NGS)	25–102^+^ y.o. (n = 12)	+/+++	+++	+++	Chromatin remodeling with age (signal differences and ↓ variance), different signals and ↓ variance in unhealthy vs. healthy centenarians	Teo et al. (2019)
ccfmiRNA	miR-126-3p	RT-qPCR	22–79 y.o. (n = 372)	+++			↑ with age	Ameling et al. (2015)
			20–90 y.o. (n = 136)	+			↑ with age (<45 vs. > 75 y.o.)	Olivieri et al. (2014)
			22–111 y.o. (n = 78)	(+)		+++	↑ with age up to 99 years old, then ↓in centenarians	Accardi et al. (2022)
			24–96 y.o. (n = 60)	++			miR-126-3p contained in EVs ↑ with age	Lo Curto et al. (2021)
	miR-30c-5p, miR-142-3p	RT-qPCR	22–79 y.o. (n = 372)	+++			↑ with age	Ameling et al. (2015)
	miR-30b-5p, miR-26a-5p, let-7a-5p			++			↑ with age	
	miR-23b-5p, miR-151a-5p			+			↑ with age	
	miR-93-5p, miR-25-5p, miR-101-3p			+			↓with age	
	miR-21	RT-qPCR	22–79 y.o. (n = 372)	++			↑ with age	
			20–105 y.o. (n = 111); centenarians’ offspring (n = 15); patients with cardiovascular disease (n = 34)	+	++	+	↑ with age, ↓ in centenarians, ↓ in centenarians’ offsprings compared to age-matched controls. ↑ in patients with cardiovascular disease compared to age-matched controls	Olivieri et al. (2012)
			30–50 y.o. healthy controls (n = 20), healthy centenarians (n = 14)			++	↓in centenarians	Balzano et al. (2017)
			22–111 y.o. (n = 78)	+		+	↑ with age up to 99 years old, then ↓in centenarians	Accardi et al. (2022)
	miR-181a	RT-qPCR	65–97 y.o. (n = 244)	++ (♂)	+++ (♀); + (♂)		↑ with age in males, but no difference with age in females. Correlates with multimorbidity in both genders	Iannone et al. (2022)
	miR-425-5p	RT-qPCR	30–50 y.o. healthy controls (n = 20), healthy centenarians (n = 14)			++	↓in centenarians	Balzano et al. (2017)
	miR-212-3p					+	↓in centenarians	
	miR-19a-3p/miR-19b-3p	Small-RNA sequencing (NGS) and RT-qPCR	Sequencing: young (25 ± 0.5 y.o., n = 3), old (71 ± 1.6 y.o., n = 3), centenarians (101.8 ± 1.1 y.o., healthy n = 3, unhealthy n = 3)	+	+	++	↑ with age, ↓ in centenarians, ↓ in healthy centenarians compared to unhealthy centenarians	Morsiani et al. (2021)
			RT-qPCR: young (30.1 ± 3.4 y.o., n = 16), old (70.8 ± 1.9 y.o., n = 16), centenarians (101.4 ± 1.3 y.o., healthy n = 11, unhealthy n = 6)	+++	+	++		

^1^“(+)” indicates an observed tendency that did not reach significance. “+” indicates a *p* < 0.05 or a significance mentioned without giving the *p-value*. “++” and “+++” indicate a *p* < 0.01 and *p* < 0.001, respectively. “/” refers to different *p*-values from different comparisons.

^2^This study was performed on serum samples.

### 3.1 Total and nuclear ccfDNA

As ccfnDNA is far more abundant in quantity than ccfmtDNA in plasma (Meddeb et al., 2019), investigations based on total ccfDNA could be considered as mainly analyzing ccfnDNA and were grouped with those focusing specifically on ccfnDNA. In a first study, Meddeb *et al* assessed ccfDNA levels during aging in plasmas of two groups of healthy subjects based on age [<and ≥47 years old (y.o.)] and showed a significant higher level of nuclear ccfDNA in the oldest group (Meddeb et al., 2019). Senescence and cell death were mainly proposed to induce higher inflammation in older adults, causing higher release of ccfDNA in plasma. Other studies characterizing plasma ccfDNA in healthy adults, including elderly, highlighted a positive correlation between age and total/nuclear ccfDNA concentrations in healthy nonagenarians compared to younger healthy controls aged between 22 and 37 years (Jylhava et al., 2011; Jylhava et al., 2013). Moreover, Tosevska et al. (2016), reported a significant decrease in ccfDNA concentrations after 6 months of exercise and nutritional supplementation, although no correlation in ccfDNA levels was found with age at baseline in three elderly groups (65–98 y.o.), suggesting that good health conditions in aging individuals could be correlated with decreased ccfDNA quantities.

Additionally, several other investigations aimed to underline the relationship between healthy aging and ccfDNA quantities. Thus, a positive correlation between ccfDNA levels and inflammatory status measured from CRP (C-reactive protein) and IL-1 (interleukin-1) levels was identified in plasmas from participants of the Vitality 90+ study, including notably 258 nonagenarians. Using a Cox regression model, ccfDNA quantitative changes were shown to be an independent determinant for all-cause mortality (Jylhava et al., 2012; Jylhava et al., 2013). These elevated concentrations of ccfDNA were also correlated with a global lower cognition status based notably on memory, perception and motor skills (Nidadavolu et al., 2022). Their conclusions went even further, indicating a risk of a decreased global cognitive score and an increased frailty score based on high amounts of ccfDNA. These results indicate that lower levels of ccfDNA could be considered as a biomarker of healthy aging in the elderly, associated to a lower risk of developing age-related pathologies.

### 3.2 ccfmtDNA

There is a growing interest in the study of ccfmtDNA as a biomarker in many diseases and an increasing number of studies have analyzed variations in ccfmtDNA in diverse biological contexts. Some of these studies have specifically focused on the analysis of ccfmtDNA during aging in healthy adults. The ccfmtDNA level was evaluated during aging in a cohort of 78 sera from healthy participants aged between 20 and 69 y.o. (Padilla-Sanchez et al., 2020). The results revealed that older subjects (≥30 y.o.) presented significantly higher levels of ccfmtDNA than younger healthy controls (≤26 y.o.). However, these differences were not found by Meddeb et al. (2019) who did not observe a significant impact of age on plasma ccfmtDNA amounts in two groups of healthy individuals: younger (<47 y.o.) and older (≥47 y.o.), despite a slightly higher ccfmtDNA copy number in the latter group. When focusing specifically on ccfmtDNA enclosed in plasma EV, a significant decrease in ccfmtDNA levels was observed with age in a cohort of middle-aged healthy subjects (30–64 y.o.) (Lazo et al., 2021).

The level of ccfmtDNA has also been analyzed throughout life, from newborns to centenarians, as well as in relation to the inflammatory status of the elderly. Thus, Pinti et al. (2014) showed an overall increase in ccfmtDNA levels with age in plasmas from individuals aged from 1 to 104 y.o., reaching its maximum at age 90. This increase in ccfmtDNA was significant since the fifth decade of life, after a slight decrease between 1 and 41 y.o. Subjects over 90 y.o. with the lowest and highest amounts of ccfmtDNA also exhibited the lowest and highest amounts of TNFα, IL-6, IL-1ra and RANTES, respectively, suggesting a correlation between ccfmtDNA and pro-inflammatory cytokines (Pinti et al., 2014). These observations led the authors to propose that ccfmtDNA could be a potential new source of inflammatory stimuli maintaining inflammaging in the elderly. Low levels of ccfmtDNA could thereby be associated to low concentrations of pro-inflammatory cytokines and inflammaging and potentially also to healthy aging.

### 3.3 ccfDNA epigenetics

ccfDNA also bears some epigenetics information, including DNA methylation and chromatin remodeling, which have been investigated in the context of aging and healthy aging. Global ccfDNA methylation was evaluated using *LINEs* and *Alu* retroelements in plasma samples of individuals aged between 18 and 64 years (Erichsen et al., 2018). DNA methylation of both repeat elements was shown to decrease significantly in the older group (41–64 y.o.) compared to the younger one (18–40 y.o.). The global hypomethylation of ccfDNA could thereby be considered as a biomarker of aging, reflecting genome instability occurring during aging (Heyn et al., 2012). Nucleosome landscape of plasma ccfDNA has also been investigated by whole genome sequencing during aging in 12 adults aged from 25 to 102^+^ years with different health conditions (Teo et al., 2019). A modification of the nucleosome landscape was described during aging accompanied by a redistribution of heterochromatin and the loss and gain of several genomic locations. Moreover, an increase from tissue ccfDNA was also highlighted in unhealthy centenarians compared to healthy centenarians, suggesting that chromatin changes profiled from ccfDNA could be used as biomarker of the health status in the elderly.

## 4 ccfmiRNAs in aging, healthy aging and longevity

MicroRNAs are small non-coding RNA around 22 nucleotides that play an important role in the modulation of gene expression at the post-transcriptional stage by targeting mRNA. They can be found in different body fluids, including blood plasma (He et al., 2020). Some studies described their differential expression during aging and/or in relation to healthy aging and longevity (Table 1).

Multiple ccfmiRNAs from plasma have been associated with aging. In a population-based study, Ameling et al. (2015) compared ccfmiRNAs levels of 372 participants from 22 to 79 y.o. and investigated associations with different phenotypes, including age. After adjustment for sex, body mass index and blood cell parameters, 12 miRNAs were significantly associated with age, including miR-126-3p, miR-30c-5p and miR-142-3p that presented the strongest association (corrected *p-value* < 0.001) with age. miR-126-3p, miR-30c-5p, miR-142-3p, miR-30b-5p, miR-26a-5p, miR-23b-5p, miR-21-5p, miR-151a-5p and let-7a-5p expression levels were found to increase with age, while those of miR-93-5p, miR-25-5p and miR-101-3p decreased with age.

Overlapping results for miR-126-3p were also reported by other studies. In a cohort of healthy subjects aged from 20 to 90 years, miR-126-3p level was significantly higher in oldest people (Olivieri et al., 2014). Measurement of miR-126-3p levels in human endothelial cells cultured *in vitro* also showed that this increase was significantly more important in senescent cells compared to young cells. Moreover, Lo Curto et al. (2021) compared levels of miR-126-3p contained in small EV (about 130 nm) in healthy subjects divided in three age classes: young (24–44 years), middle-aged (53–68 years) and old (81–96 years). After plasma isolation and small EV purification by ultra-centrifugation, miRNAs were quantified by RT-qPCR assays. The level of miR-126-3p contained in EVs increased with age, in agreement with previously obtained results. Thus, miR-126-3p could be considered as a circulating biomarker of senescence in the elderly.

In a study conducted on a cohort of 224 participants from 65 to 97 y.o., another ccfmiRNA, miR-181a, has also been associated with aging (Iannone et al., 2022). Higher level of this miRNA was found in older men compared to younger ones, but no differences were observed in women. The authors showed that circulating miR-181a levels correlated with multimorbidity, and suggested that its high expression is associated with high inflammation. Of note, miR-181a played an important role in inflammation, modulating anti-inflammatory TGF-β and IL-10, and pro-inflammatory cytokines such as IL-1, IL-6 and TNFα (Xie et al., 2013). It could thereby be directly implicated in the aging process and be a potential biomarker of the health status.

Several studies have further investigated the potential of ccfmiRNAs as biomarkers of aging, healthy aging and/or longevity relying specifically on the inclusion of healthy nonagenarians and/or centenarians in their study design. By comparing 7 healthy individuals aged 20, 80 and 100 y.o., 365 ccfmiRNA expression profiles were identified and classified according to their highest expression in each of the three age groups (Olivieri et al., 2012). After validation with a control cohort of 111 healthy participants aged between 20 and 105 y.o., miR-21 was shown to have a significantly higher expression in older people (66–96 y.o.) compared to the younger group (20–65 y.o.) and centenarians. However, the study did not find any significant differences in miR-21 expression between centenarians and the youngest group. Additionally, compared to age-matched controls, a lower level of miR-21 in centenarians’ offspring and a higher expression in cardiovascular patients were highlighted (Olivieri et al., 2012). miR-21 could be implicated in inflammaging and affect the risk of age-related diseases. It could negatively modulate the TGF-β pathway, resulting in an increased inflammation (Olivieri et al., 2012). In another study, Balzano *et al.* identified a reduced level of miR-21, alongside miR-425-5p and miR-212-3p, in plasma samples of 14 healthy centenarians compared with 20 healthy controls from 30 to 50 y.o. (Balzano et al., 2017). Like miR-21, miR-425-5p is overexpressed during inflammation, and the authors speculated that a low inflammation level might help centenarians to survive to age-related diseases (Balzano et al., 2017). Moreover, the low expression of both miR-21 and miR-425-5p should lead to an increased *PTEN* (phosphatase and tensin homolog) expression, a tumor suppressor gene also known to be a promoter of longevity (Ortega-Molina and Serrano, 2013; Balzano et al., 2017). Similar results regarding miR-21 and also miR-126-3p were found in a Sicilian cohort of 78 healthy controls from 22 to 111 y.o. The authors described their increased expression with age that decreased in centenarians, suggesting that both ccfmiRNAs could be biomarkers of longevity (Accardi et al., 2022).

Recently, circulating miR-19a-3p and miR-19b-3p have also been associated with aging, healthy aging and longevity (Morsiani et al., 2021). In a small cohort of 12 participants with different ages classified into young (mean age = 25 y.o.), old (mean age = 71 y.o.), healthy and unhealthy centenarians, small-RNA sequencing experiments showed that expression levels of these two miRNAs increased at old age compared to younger subjects, and decreased in centenarians; a finding that was further validated in a cohort of 49 plasma samples. Sequencing data also exhibited a significant decrease of both ccfmiRNAs in healthy centenarians compared to unhealthy centenarians, albeit not validated in the replication cohort. These observed differences were attributed to the presence of isomiRs, with isomiR-19a-3p differing significantly in both groups of centenarians. As miR-19a-3p and miR-19b-3p regulate *PTEN* expression and are also involved in two pathways (FoxO and IGF-mTor) associated with aging and longevity, both miRNAs could be considered as potential longevity and (healthy) aging biomarkers (Ortega-Molina and Serrano, 2013; Morsiani et al., 2021).

## 5 Concluding remarks and perspectives

We have summarized the current state of knowledge on plasma ccfNAs during aging and in relation to healthy aging and longevity. Concerning ccfDNA, the mentioned studies strongly support the idea that ccfDNA could serve as a biomarker of (healthy) aging at genetic and epigenetic level. An increase in ccfDNA concentration, a global ccfDNA hypomethylation as well as a reorganization of heterochromatin inferred from ccfDNA nucleosome signals were observed during aging. In nonagenarians and/or centenarians, lower levels of ccfDNA was associated with reduced inflammation and a better health status, while higher levels increased the risk of frailty and cognitive decline. Moreover, ccfmtDNA was also correlated to several inflammatory cytokines (TNFα, IL-6, IL-1ra and RANTES) and was suggested as a potential mediator of inflammation. Therefore, ccfDNA could be considered as a biomarker of healthy aging and of the inflammatory status in the elderly. In addition, several plasma ccfmiRNAs were identified as biomarkers of aging (Table 1), some of which being also associated with healthy aging and longevity (miR-21 and miR-126-3p) or with inflammation (miR-21 and miR-181a). The identification of some ccfmiRNA (miR-21, miR-425-5p and miR-19a/b-3p) in healthy centenarians, which are implicated in the FoxO and IGF-mTor pathways and/or the regulation *PTEN* expression, also support the hypothesis of a possible biological role of these miRNAs in aging and longevity.

Perspectives for ccfNAs in the field are numerous and should encompass both fundamental and translational aspects. Basic investigations could focus on the characterization of the putative functions of ccfNAs in aging and longevity, especially for ccfmtDNA and ccfmiRNAs. They could aim to decipher the molecular mechanisms by which ccfmtDNA could directly modulate the production of proinflammatory cytokines and therefore contribute to inflammaging, as well as how specific ccfmiRNAs affect aging and longevity. Translational researches could include the validation and evaluation of the predictive performances of ccfNAs identified as healthy aging biomarkers in replication studies on large longitudinal or experimental cohorts. We can also speculate about the future development of combined panels of ccfNAs-based biomarkers, including ccfDNA and ccfmiRNAs, that could be used to predict healthspan and lifespan as well as various risks and diseases in the elderly. Moreover, ccfmiRNAs associated with longevity might represent some potential bioactive molecules for healthy aging and/or anti-aging interventions, particularly when enclosed in EV (micro-vesicles and exosomes).
